# Preclinical comparison of four [^18^F, ^nat^Ga]rhPSMA-7 isomers: influence of the stereoconfiguration on pharmacokinetics

**DOI:** 10.1186/s13550-020-00740-z

**Published:** 2020-12-07

**Authors:** Alexander Wurzer, Mara Parzinger, Matthias Konrad, Roswitha Beck, Thomas Günther, Veronika Felber, Stefanie Färber, Daniel Di Carlo, Hans-Jürgen Wester

**Affiliations:** Chair of Pharmaceutical Radiochemistry, Technical University of Munich, Walther-Meißner-Str. 3, 85748 Garching, Germany

**Keywords:** PSMA, Fluorine-18, Prostate cancer, Radiohybrid

## Abstract

**Introduction:**

Radiohybrid (rh) ligands, a novel class of prostate-specific membrane antigen (PSMA)-targeted radiopharmaceuticals, can be labeled either with [^18^F]fluorine via isotopic exchange or with radiometals (such as [^68^Ga]Gallium, [^177^Lu]Lutetium, [^225^Ac]Actinium). Among these, [^18^F, ^nat^Ga]rhPSMA-7 has recently entered clinical assessment.

**Aim:**

Since [^18^F, ^nat^Ga]rhPSMA-7 is composed of four stereoisomers ([^18^F, ^nat^Ga]rhPSMA-7.1, -7.2, -7.3 and -7.4), we initiated a preclinical selection process to identify the isomer with the most favorable pharmacokinetics for further clinical investigation.

**Methods:**

A synthetic protocol for enantiopure [^19^F, ^nat^Ga]rhPSMA-7 isomers has been developed. The comparative evaluation of the four isomers comprised human serum albumin binding, lipophilicity, IC_50_, internalization and classical biodistribution studies and competition experiments in LNCaP tumor-bearing CB-17 SCID mice. In addition, a radio high-performance liquid chromatography-based method was developed allowing quantitative, intraindividual comparison of [^18^F, ^nat^Ga]rhPSMA-7.1 to -7.4 in LNCaP tumor-bearing mice.

**Results:**

Cell studies revealed high PSMA affinity and internalization for [^18/19^F, ^nat^Ga]rhPSMA-7.2, -7.3 and -7.4, whereas [^18/19^F, ^nat^Ga]rhPSMA-7.1 showed approximately twofold lower values. Although the biodistribution profile obtained was typical of PSMA inhibitors, it did not allow for selection of a lead candidate for clinical studies. Thus, an intraindividual comparison of all four isomers in LNCaP tumor-bearing mice was carried out by injection of a diastereomeric mixture, followed by analysis of the differential uptake and excretion pattern of each isomer. Based on its high tumor accumulation and low uptake in blood, liver and kidneys, [^18^F, ^nat^Ga]rhPSMA-7.3 was identified as the preferred isomer and transferred into clinical studies.

**Conclusion:**

[^18^F, ^nat^Ga]rhPSMA-7.3 has been selected as a lead compound for clinical development of a [^18^F]rhPSMA-based candidate. The intraindividual differential uptake and excretion analysis in vivo allowed for an accurate comparison and assessment of radiopharmaceuticals.

## Introduction

During the last decade, advancements in the field of prostate-specific membrane antigen (PSMA)-targeting radiopharmaceuticals have had significant impact on the clinical management of patients suffering from prostate cancer [1–3]. [^68^Ga]Ga-PSMA-11 [4, 5] in particular has been the subject of extensive evaluation and has already proved its eligibility, especially for positron emission tomography (PET)-based detection of biochemical recurrence [6, 7], resulting in its broad clinical use [8, 9].

Due to the superior nuclear properties of the fluorine-18 radionuclide and accompanying logistic and economic advantages [10, 11], a shift of interest from ^68^Ga-labeled PSMA tracers toward ^18^F-labeled analogues has been observed in recent years [12–14]. In this context radiohybrid (rh) PSMA ligands, developed by our group, form a novel class of radiopharmaceuticals, which combine a Silicon–Fluoride–Acceptor (SiFA) and a metal chelate (or a chelator) in a single molecule [15]. Such rhPSMA ligands can either be labeled with fluorine-18 by isotopic exchange at the SiFA-moiety in the presence of a non-radioactive metal chelate (e.g., ^nat^Ga- or ^nat^Lu-chelate), or with a radiometal (e.g., [^68^Ga]Gallium, [^177^Lu]Lutetium, [^225^Ac]Actinium) by means of the chelator, while the SiFA moiety is non-radioactive [15]. The chemical identity of the ^18^F-labeled non-radioactive metal complexed radiohybrid ligand (**[**^**18**^**F, **^**nat**^**M]ligand**) with the radio-metallated fluorine-19 compound (**[**^**19**^F, *M]ligand) offers unique options for imaging and theranostic applications. [^18^F, ^nat^Ga]rhPSMA-7 (often abbreviated as [^18^F]rhPSMA-7) has already been assessed for PET imaging of primary and metastatic castration-resistant prostate cancer. Biodistribution of [^18^F, ^nat^Ga]rhPSMA-7 was found to be similar to that of established PSMA ligands, and [^18^F, ^nat^Ga]rhPSMA-7 PET/CT demonstrated high detection rates in early biochemical recurrence after radical prostatectomy, especially among patients with low prostate-specific antigen values [16]. Furthermore, the novel tracer outperformed morphologic imaging for N-staging of high-risk primary prostate cancer, with efficacy comparable to the literature data for [^68^Ga]Ga-PSMA-11 [17]. The highest imaging quality of this tracer was obtained at 50–70 min post injection in PET/CT [18].

**Fig. 1:**
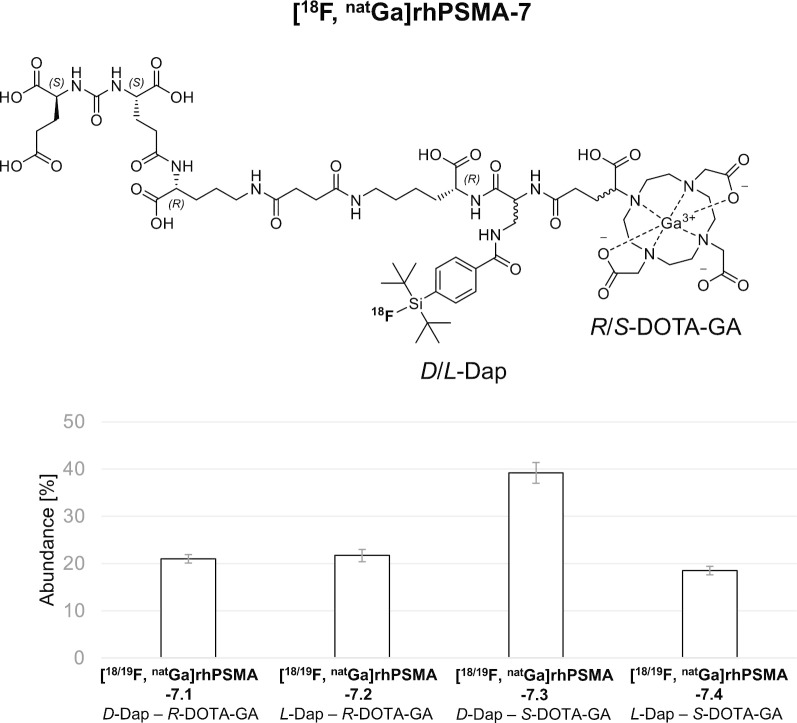
Diastereomeric mixture [^18^F, ^nat^Ga]rhPSMA-7 is composed of the four isomers [^18^F, ^nat^Ga]rhPSMA-7.1 to -7.4, differing in the stereoconfiguration of diaminopropionic acid (*D-*/*L*-Dap) and DOTA-GA (*R*-/*S*-DOTAGA). The abundance of the diastereomers could be determined by HPLC-analysis of retained samples of previously produced batches of the diastereomeric mixture, [^19^F, ^nat^Ga]rhPSMA-7 (n = 6)

[^18^F, ^nat^Ga]rhPSMA-7 represents a mixture of four stereoisomers (Fig. 1), differing in the stereoconfiguration of the diaminopropionic acid branching unit (*D*-Dap or *L*-Dap) and the glutamic acid pendant arm at the DOTA-GA-chelator (*R*-DOTA-GA or *S*-DOTA-GA; DOTA-GA: 2-(4,7,10-tris(carboxymethyl)-1,4,7,10-tetraazacyclododecan-1-yl)pentanedioic acid). After the diastereomeric mixture [^18^F, ^nat^Ga]rhPSMA-7 has been successfully assessed in a clinical setting in > 1000 prostate cancer patients, we initiated a selection process in order to identify the stereoisomer with the most promising characteristics for further clinical studies.

Here, we report a preclinical comparison of the four rhPSMA-7 isomers, [^18^F, ^nat^Ga]rhPSMA-**7.1** (*D*-Dap–*R*-DOTA-GA), [^18^F, ^nat^Ga]rhPSMA-**7.2** (*L*-Dap–*R-*DOTA-GA), [^18^F, ^nat^Ga]rhPSMA-**7.3** (*D*-Dap–*S*-DOTA-GA) and [^18^F, ^nat^Ga]rhPSMA-**7.4** (*L*-Dap–*S*-DOTA-GA), prepared by means of a novel synthetic procedure. The isomers were evaluated in vitro (IC_50_, binding to and internalization into LNCaP cells, binding to human serum albumin (HSA)) and biodistribution studies in LNCaP tumor-bearing SCID mice were performed and compared to the diastereomeric mixture [^18^F, ^nat^Ga]rhPSMA-7. Aim of this study was to identify the isomer with the most favorable pharmacokinetics for further clinical development.

## Materials and methods

### General information

CheMatech (Dijon, France) delivered *R-* and *S-*configured protected DOTA-GA chelators. (*S*)-5-(*tert*-butoxy)-4-(3-((*S*)-1,5-di-*tert*-butoxy-1,5-dioxopentan-2-yl)ureido)-5-oxopentanoic acid ((*t*BuO)EuE(O*t*Bu)_2_) and the SiFA-moiety, 4-(di-*tert*-butylfluorosilyl)benzoic acid were prepared according to the literature protocols [19, 20]. Conjugation reagents for solid-phase peptide synthesis (SPPS) were either HOBt (1-Hydroxybenzotriazole), TBTU (*N*-[(*1H*-Benzotriazol-1-yl)(dimethylamino)-methylene]-*N*-methylmethanaminium-tetrafluoroborate-*N*-oxide) or HATU (*N*-[(7-Aza-*1H*-benzotriazol-1-yl)(dimethylamino)-methylene]-*N*-methylmethanaminium-hexafluorophosphate-*N*-oxide) together with DIPEA (diisopropylethylamine) or 2,4,6-trimethylpyridine. PSMA-positive LNCaP cells were purchased from Cell Lines Service (Eppelheim, Germany).

Analytical and preparative high-performance liquid chromatography (HPLC) was performed using Shimadzu gradient systems (Kyoto, Japan) equipped with a SPD-20A UV/Vis detector. The columns for analytical (Nucleosil 100C18, 125 × 4.6 mm, 5 μm), radio-analytical (Multospher 100RP18, 125 × 4.6 mm, 5 μm) and preparative (Multospher 100RP18, 250 × 10 mm, 5 μm) HPLC were purchased from CS Chromatographie Service (Langerwehe, Germany). Eluents for all HPLC operations were water (solvent A) and acetonitrile (solvent B), both containing 0.1 vol.% trifluoroacetic acid (TFA). Radioactivity was detected via a HERM LB 500 NaI detector and a Flowstar^2^ LB514 detector (Berthold Technologies, Bad Wildbad, Germany). Electrospray ionization-mass spectra were acquired on an expression^L^ CMS (Advion, Harlow, UK).

### Chemical synthesis

The four isomers ([^19^F, ^nat^Ga]rhPSMA-7.1–7.4) were prepared via SPPS by means of modifying the recently published protocol for diastereomeric mixture [^19^F, ^nat^Ga]rhPSMA-7 (see Additional file 1: Figs. 1 to 5) [15].

### Radiolabeling

Manual ^18^F-labeling.

[^18^F]Fluoride (approx. 0.6–2.0 GBq/mL) was provided by the Klinikum rechts der Isar (Munich, Germany). Non-automated ^18^F-labeling was conducted as described previously [15].

#### Automated ^18^F-labeling

The production of [^18^F, ^nat^Ga]rhPSMA-7 with starting activities of 50–100 GBq was performed by means of a fully-automated procedure at the Klinikum rechts der Isar (see Additional file 1: Fig. 6) [15].

### Lipophilicity and binding to human serum albumin

Approximately 1 MBq of the ^18^F-ligand was dissolved in 1 mL of a 1:1 mixture (*v*/*v*) of phosphate-buffered saline (PBS, pH 7.4) and *n*-octanol (n = 18–23). After vigorous mixing of the suspension for 3 min, the vial was centrifuged at 15,000 × g for 3 min and 100 μL aliquots of both layers were measured in a γ-counter. HSA binding of ^19^F-^nat^Ga-rhPSMA ligands was determined according to a previously published procedure [15, 21].

### In vitro experiments

#### Affinity determinations (IC_50_) and internalization studies

Competitive binding studies were determined on LNCaP cells (1.5 × 10^5^ cells in 1 mL/well) after incubation at 4 °C for 1 h, using (((*S*)-1-carboxy-5-(4-([^125^I]iodo)benzamido)pentyl)carbamoyl)-*L*-glutamic acid (([^125^I]I-BA)KuE; 0.2 nM/well) as reference radioligand (n = 5–9). Internalization studies of the radiolabeled ligands (0.5 nM/well) were performed on LNCaP cells (1.25 × 10^5^ cells in 1 mL/well) at 37 °C for 1 h and accompanied by ([^125^I]I-BA)KuE (0.2 nM/well), as reference. Data were corrected for non-specific binding and normalized to the specific internalization observed for the reference (n = 3–6). A detailed description of the experimental procedures was previously published [15].

### In vivo experiments

All animal experiments were conducted in accordance with general animal welfare regulations in Germany (German animal protection act, as amended on 18.05.2018, Art. 141 G v. 29.3.2017 I 626, approval no. 55.2-1-54-2532-71-13 by the General Administration of the Free State of Bavaria) and the institutional guidelines for the care and use of animals.

LNCaP tumor xenografts were established in 6–8 weeks old male CB-17 SCID mice as described previously [15].

#### Biodistribution

1–3 MBq (0.2 nmol) of the [^18^F, ^nat^Ga]rhPSMA inhibitors were injected into the tail vein of LNCaP tumor-bearing male CB-17 SCID mice that were sacrificed 1 h post injection (p.i.) (n = 4 for 7.1, n = 5 for 7.2, n = 4 for 7.3, n = 5 for 7.4 and n = 3 for 7). Selected organs were removed, weighed, and measured in a γ-counter. For competition studies, 8 mg/kg of 2-PMPA (2-(Phosphonomethyl)-pentandioic acid) were co-administered (n = 3).

#### Differential uptake and excretion pattern

With the intention to identify even marginal differences in the in vivo behavior, all four [^18^F, ^nat^Ga]rhPSMA-7 isomers were co-evaluated in one single tumor-bearing mouse in order to quantify the differential uptake and excretion pattern of each isomer. For this purpose, the diastereomeric mixture [^18^F, ^nat^Ga]rhPSMA-7 was produced in high molar activity (A_M_) of 247–349 GBq/µmol (at end of synthesis) and the abundance of each isomer was determined by radio-HPLC. Mice (n = 4) were injected with [^18^F, ^nat^Ga]rhPSMA-7 (180–280 MBq, 0.9–1.0 nmol) and left under anesthesia. After 30 min p.i., they were sacrificed and urine, blood, liver, kidneys and tumor were collected and processed. Radioactivity was extracted from solid tissues by means of a Potter–Elvehjem tissue grinder (n = 1) or a ball mill (n = 3) and separated from the protein fraction by subsequent cartridge-based solid-phase extraction (SPE). Blood samples were diluted, centrifuged and the supernatant was purified by SPE (see supporting information). Urine and extracts from tissue samples were then analyzed by radio-HPLC to quantify the fraction of each isomer in each sample. Finally, the relative percentage difference between the abundance of each isomer in the injected diastereomeric mixture (values taken from the quality control) and the abundance of the respective isomer in each analyzed tissue or body fluid was calculated. To accurately quantify the abundance of the isomers, especially in cases when no baseline separation was achieved in radio-HPLC, all radio-HPLC profiles were processed by the Systat (San Jose, US) software package PeakFit. PeakFit allows for automated nonlinear separation, analysis and quantification of HPLC elution profiles by deconvolution procedures that uses a Gaussian response function with a Fourier deconvolution/filtering algorithm (see Additional file 1: Fig. 7).

## Results

### Chemical synthesis

When employing standard coupling conditions (HOBt, TBTU and DIPEA) during the initial production of [^19^F, ^nat^Ga]rhPSMA-7, conversion of *D*- to *L*-Dap was observed. Optimization was carried out by either substitution of the coupling reagents HOBt and TBTU by HATU or replacement of DIPEA by the weaker base 2,4,6-trimethylpyridine. Whereas the use of HATU already reduced the conversion to about 12%, it was almost eliminated (< 2%) using 2,4,6-trimethylpyridine.

To eliminate the second source of isomers, racemic DOTA-GA employed in [^19^F, ^nat^Ga]-rhPSMA-7 was substituted by the enantiomerically pure *S*- and *R*-DOTA-GA. After non-radioactive metal complexation with Ga(NO_3_)_3_ and final purification, HPLC analysis (UV absorbance at 220 nm) revealed > 98% purity for all ^19^F-^nat^Ga-rhPSMA-7 isomers and also confirmed the absence of undesired diastereomers.

### Analytical characterization

Previously produced clinical batches of diastereomeric [^19^F, ^nat^Ga]rhPSMA-7 (n = 6) were analyzed by HPLC to retrospectively determine the abundance of the individual isomers [^19^F, ^nat^Ga]rhPSMA-7.1 to -7.4. Since insufficient peak separation was obtained, employing standard HPLC conditions (10–70 vol.% acetonitrile/ 90–30 vol.% water in 15 min, both solvents supplemented with 0.1 vol.% TFA, 1.0 mL/min), the gradient was optimized. Best separation conditions were found with a gradient of 25–35% acetonitrile/water (25–35 vol.% acetonitrile/ 75–65 vol.% water, both solvents supplemented with 0.1 vol.% TFA, 1.0 mL/min) in 40 min (see Additional file 1: Fig. 8). Peak assignment was carried out by comparison of the HPLC profile of the diastereomeric mixture [^19^F, ^nat^Ga]rhPSMA-7 without and with co-injection of each enantiopure isomer. [^19^F, ^nat^Ga]rhPSMA-7.3 was found to be the dominant species (39%), while the other isomers [^19^F, ^nat^Ga]rhPSMA-7.1, -7.2 and -7.4 were present in similar relative amounts (18–22%); Fig. 1.

### Radiolabeling

Drying of aqueous [^18^F]fluoride followed by ^18^F-labeling by ^18^F-for-^19^F isotopic exchange was carried out as previously described [15, 22]. [^18^F, ^nat^Ga]rhPSMAs were obtained in 60 ± 10% radiochemical yield (RCY) and a A_M_ of up to 60 GBq/µmol (starting with 2–5 GBq [^18^F]fluoride and 50–100 nmol [^19^F, ^nat^Ga]rhPSMA-7.x) within 20 min. As determined by HPLC and thin-layer chromatography (TLC), [^18^F, ^nat^Ga]rhPSMA-7.1 to -7.4 were generally obtained in high radiochemical purity (RCP > 97%).

Automated ^18^F-labeling of the diastereomeric mixture [^19^F, ^nat^Ga]rhPSMA-7 (150 nmol) was performed by means of a fully-automated procedure at the Klinikum rechts der Isar (50–100 GBq starting activity) in 15 min; RCY of 50 ± 8%, A_M_ up to 300 GBq/µmol (RCP > 97% by HPLC and TLC).

### In vitro characterization

**Fig. 2:**
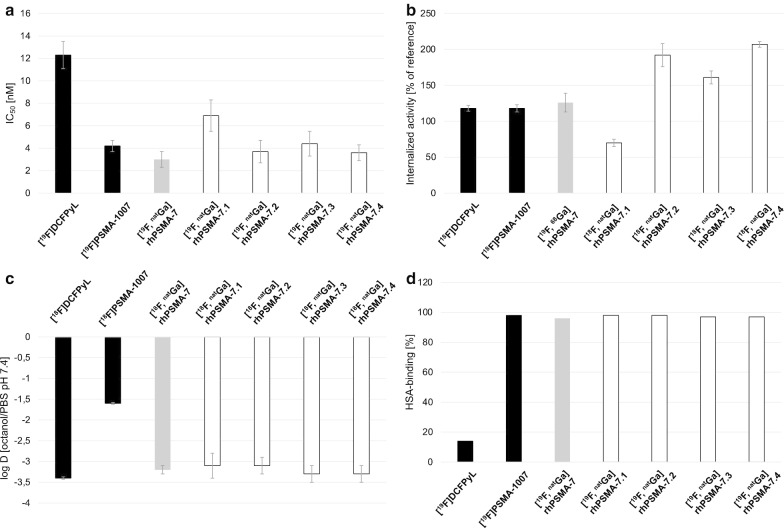
**a** Binding affinities (IC_50_ in nM, 1 h, 4 °C) of [^19^F, ^nat^Ga]rhPSMA-7.1 to -7.4 (white; n = 5–9) and the references diastereomeric mixture [^19^F, ^nat^Ga]rhPSMA-7 (grey; n = 3), [^19^F]DCFPyL and [^19^F]PSMA-1007 (black; n = 3); **b** internalized activity of [^18^F, ^nat^Ga]rhPSMA-7.1 to -7.4 (white; n = 3–6) and the references diastereomeric mixture [^19^F, ^68^ Ga]rhPSMA-7 (grey; n = 3), [^18^F]DCFPyL and [^18^F]PSMA-1007 (black; n = 3) in LNCaP cells (1 h, 37 °C) as a percentage of the reference ligand ([^125^I]I-BA)KuE); **c** lipophilicity of [^18^F, ^nat^Ga]rhPSMA-7.1 to -7.4 (white; n = 18–23) and the references diastereomeric mixture [^18^F, ^nat^Ga]rhPSMA-7 (grey; n = 13), [^18^F]DCFPyL and [^18^F]PSMA-1007 (black; n = 3), expressed as *n*-octanol/PBS (pH 7.4) distribution coefficient (log D); **d** human serum albumin binding of [^19^F, ^nat^Ga]rhPSMA-7.1 to -7.4 (white) and the references diastereomeric mixture [^19^F, ^nat^Ga]rhPSMA-7 (grey), [^19^F]DCFPyL and [^19^F]PSMA-1007 (black), determined on a Chiralpak HSA column. Data for the reference ligands were [15, 19] taken from a previously published studies conducted by our group. Values are expressed as mean ± standard deviation

In vitro data obtained with the four [^18/19^F, ^nat^Ga]rhPSMA-7 isomers are summarized in Fig. 2 and Additional file 1: Table 1; data from the references [^18/19^F]DCFPyL [23, 24], [^18/19^F]PSMA-1007 [25, 26] and [^18/19^F, ^nat^Ga]rhPSMA-7, evaluated under identical experimental conditions, were taken from previously published studies by our group and are included for comparison [15, 19].

The PSMA binding affinities of the isomers [^19^F, ^nat^Ga]rhPSMA-7.2, -7.3 and -7.4 to LNCaP cells (IC_50_; Fig. 2a: 3.7 ± 1.0 nM; 4.4 ± 1.1 nM; 3.6 ± 0.7 nM, respectively) were found to be comparable to that of diastereomeric mixture [^19^F, ^nat^Ga]rhPSMA-7 (IC_50_:3.0 ± 0.7 nM) and [^19^F]PSMA-1007 (IC_50_:4.2 ± 0.5 nM). In contrast, the *D*-Dap–*R*-DOTA-GA-isomer [^19^F, ^nat^Ga]rhPSMA-7.1 showed a slightly reduced affinity (IC_50_:6.9 ± 1.4 nM), which was, however, still 1.8-fold higher than that of [^19^F]DCFPyL (IC_50_:12.3 ± 1.2 nM).

In addition, [^18^F, ^nat^Ga]rhPSMA-7.2 to -7.4 also showed improved internalization (Fig. 2b: 192 ± 16%, 161 ± 9% and 207 ± 4%, respectively) at 1 h, 37 °C, whereas internalization of [^18^F, ^nat^Ga]rhPSMA-7.1 (70 ± 5%) was even lower than those of the reference compounds [^18^F]DCFPyL (118 ± 4%) and [^18^F]PSMA-1007 (118 ± 5%).

The lipophilicity determined for the diastereomeric mixture and the four isomers was almost identical (mixture: log D = -3.2 ± 0.1; isomers: log D = -3.1 ± 0.3 to -3.3 ± 0.2). A similar distribution coefficient has previously been determined for [^18^F]DCFPyL (log D = -3.4 ± 0.03), whereas [^18^F]PSMA-1007 (log D = -1.6 ± 0.02) was found to be more lipophilic (Fig. 2c).

While binding of [^19^F, ^nat^Ga]rhPSMA-7.1 to -7.4 and [^19^F]PSMA-1007 to HSA was found to be strong (> 96%), [^19^F]DCFPyL showed only very low binding to HSA (14%), (Fig. 2d) [15, 21].

### Biodistribution studies

**Fig. 3:**
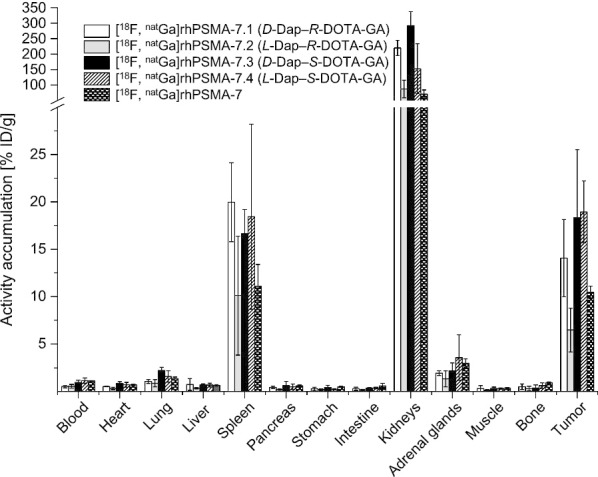
Biodistribution of [^18^F, ^nat^Ga]-rhPSMA-7 and the isomers [^18^F, ^nat^Ga]rhPSMA-7.1 to -7.4 at 1 h p.i. in male LNCaP tumor-bearing SCID mice. Data are expressed as a percentage of the injected dose per gram (% ID/g), mean ± standard deviation (n = 4 for [^18^F, ^nat^Ga]rhPSMA-7.1, n = 5 for -7.2, n = 4 for -7.3, n = 5 for -7.4 and n = 3 for -7)

Biodistribution studies at 1 h p.i. revealed a biodistribution pattern of [^18^F, ^nat^Ga]rhPSMA-7.1, -7.2, -7.3 and -7.4 quite similar to that of diastereomeric mixture [^18^F, ^nat^Ga]rhPSMA-7 (Fig. 3 and Additional file 1: Table 2). Differences between individual blood clearance kinetics correlate with the tissue distribution profiles. The highest tumor and kidney accumulation were found for the *S*-DOTA-GA-based isomers [^18^F, ^nat^Ga]rhPSMA-7.3 and -7.4, followed by -7.1. In contrast, the *L*-Dap–*R*-DOTA-GA ligand [^18^F, ^nat^Ga]rhPSMA-7.2 showed an approximately twofold lower tumor and kidney uptake compared with the other isomers.

### Biodistribution studies with competition

**Fig. 4:**
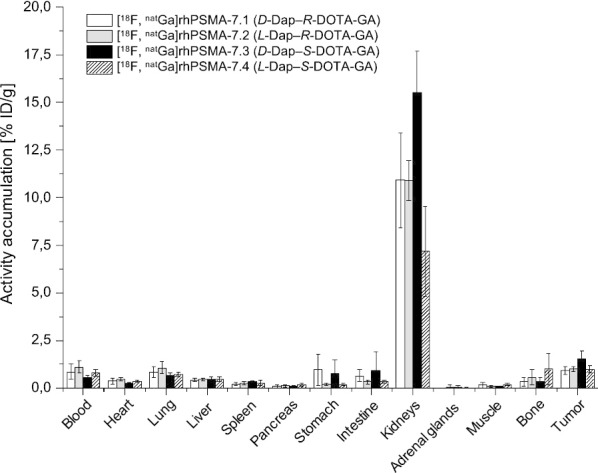
Biodistribution of [^18^F, ^nat^Ga]rhPSMA-7.1 to -7.4, co-injected with 2-PMPA (8 mg/kg) at 1 h p.i. in male LNCaP tumor-bearing SCID mice. Data are expressed as a percentage of the injected dose per gram (% ID/g), mean ± standard deviation (n = 3)

Specificity of binding was demonstrated by co-injection of the potent PSMA inhibitor 2-PMPA (8 mg/kg) with [^18^F, ^nat^Ga]rhPSMA-7.1 to -7.4 (Fig. 4 and Additional file 1: Table 3). As expected, the competition experiments resulted in highly efficient reduction of the uptake of respective isomers in kidneys, tumor, spleen and adrenal glands. Under competition, tumor uptake (0.9 ± 0.2%ID/g to 1.5 ± 0.4%ID/g) only marginally exceeded the activity concentrations in blood (0.6 ± 0.1%ID/g to 1.1 ± 0.3%ID/g) for all isomers. The normal kidney uptake (range: 88 ± 29%ID/g to 292 ± 45%ID/g) was also markedly lowered (range: 7 ± 2%ID/g to 16 ± 2%ID/g).

### Differential uptake and excretion pattern

With the aim to generate a more robust set of comparative data that might allow for a well-founded selection of the most promising isomer, we decided to co-assess [^18^F, ^nat^Ga]rhPSMA-7.1 to -7.4 intraindividually (n = 4) by analyzing the differential uptake and excretion pattern in urine, blood, liver, kidneys and tumor after injection of [^18^F, ^nat^Ga]rhPSMA-7 by means of radio-HPLC analyses.

#### Tissue extraction

The percentage of the radioactivity which was extracted from tissue samples and obtained after SPE-based removal of the protein fraction is summarized in Table 1. Most of the activity could be recovered from blood (90 ± 4%) and liver (86 ± 2%). When compared with the ball mill, sample extraction via the manual tissue grinder was slightly more efficient for kidney (91% vs. 63 ± 5%) and tumor (90% vs. 64 ± 18%). However, after both procedures a significant amount of the extracted activity could not be trapped on the SPE cartridge, resulting in a decreased overall extraction efficiency of 43–60% and 42–53% for kidney and tumor, respectively.

**Table 1:** Extraction efficiency of [^18^F, ^nat^Ga]rhPSMA-7 from blood, liver, kidney and tumor.

	Tissues	Efficiency [% Extracted radioactivity]		
		Sample extraction	SPE purification	Overall
Potter–Elvehjem tissue grinder (n = 1)	Kidney	91	66	60
	Tumor	90	59	53
MM-400 ball mill (n = 3)	Liver	97	89 ± 2	86 ± 2
	Kidney	63 ± 5	68 ± 8	43 ± 8
	Tumor	64 ± 18	65 ± 3	42 ± 14
	Blood (n = 4)	97 ± 2	94 ± 2	90 ± 4

Samples were extracted using either a Potter–Elvehjem tissue grinder (n = 1) or a MM-400 Ball Mill (n = 3). The percentage of activity after sample extraction and after SPE purification was quantified, decay-corrected, and the overall extracted activity was calculated (values are expressed as mean ± SD).

#### Metabolic stability

Radio-HPLC analyses of urine samples and of the radioactivity extracted from the homogenized (kidney, liver, tumor) or diluted (blood) samples did not show any radiolabeled fragments of [^18^F, ^nat^Ga]rhPSMA-7.

#### Differential uptake and excretion pattern

**Fig. 5:**
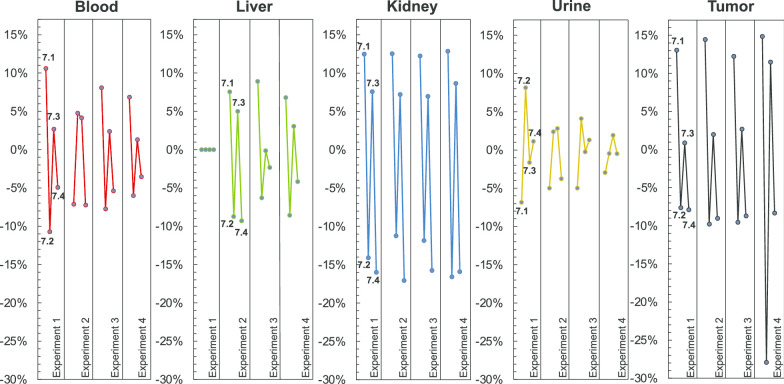
Differential uptake and excretion pattern: relative percentage difference between the abundance of [^18^F, ^nat^Ga]rhPSMA-7.1 to -7.4 in blood, liver, kidney, urine and tumor of male LNCaP tumor-bearing SCID mice (30 min p.i.) compared with the respective abundance found in the quality control of diastereomeric mixture [^18^F, ^nat^Ga]rhPSMA-7. n = 4 in four independent experiment; the liver from the first experiment was not analyzed (see also Additional file 1: Fig. 9)

The differential uptake and excretion pattern of [^18^F, ^nat^Ga]rhPSMA-7 isomers in urine, blood, liver, kidneys and tumor is shown in Fig. 5, and expressed as the relative percentage difference between the abundance of the isomer in the quality control (and thus, at the time point of injection into tumor-bearing mice) and the abundance of the isomer in the analyzed tissue or body fluid (also see Additional file 1: Fig. 9). Compared to the time point of injection, the abundance of the *D*-Dap-containing isomers [^18^F, ^nat^Ga]rhPSMA-7.1 and -7.3 was found to be higher in blood, liver, kidney and tumor samples in all experiments, thus demonstrating a stronger ´enrichment´ of *D*-Dap-based isomers when compared with the *L*-Dap-isomers [^18^F, ^nat^Ga]rhPSMA-7.2 and -7.4. One outlier was found in the second experiment of the blood sample, in which [^18^F, ^nat^Ga]rhPSMA-7.2 showed higher uptake than [^18^F, ^nat^Ga]rhPSMA-7.1. Other than that, the highest accumulation was consistently found for [^18^F, ^nat^Ga]rhPSMA-7.1, followed by [^18^F, ^nat^Ga]rhPSMA-7.3 in all solid tissues and blood. [^18^F, ^nat^Ga]rhPSMA-7.2 and -7.4 showed the lowest enrichment in blood, liver and kidney, but also the lowest uptake by tumors.

## Discussion

The novel radiohybrid PSMA-targeting ligand [^18^F, ^nat^Ga]rhPSMA-7 already demonstrated promising results in staging and restaging of prostate cancer [16, 17]. Since the compound is composed of four stereoisomers ([^18^F, ^nat^Ga]rhPSMA-7.1, -7.2, -7.3 and -7.4), this study aimed to identify the one isomer with the most promising characteristics for further clinical investigation.

Prior to the actual selection process, the chemical synthesis needed to be improved to produce the individual [^19^F, ^nat^Ga]rhPSMA-7 isomers. Suppression of unexpected conversion of *D*- to *L*-Dap was achieved by using the weaker base 2,4,6-trimethylpyridine. The benefits of 2,4,6-trimethylpyridine, particularly when used in conjunction with structurally-related cysteine analogues, have already been described in the literature [27]. The authors speculate that formation of an active-ester of Fmoc-Dap(Dde)-OH during pre-activation increases acidity of the proton at the α-carbon, which then is abstracted by DIPEA. The abstraction is also promoted by the strong electron-withdrawing effect of the Dde-protecting group, resulting in a partial positive charge at the β-amine of Dap. In contrary 2,4,6-trimethylpyridine seems to be too weak and bulky for proton abstraction.

This key step and the application of the respective enantiopure DOTA-GA chelator allowed for production of the [^19^F, ^nat^Ga]rhPSMA-7 isomers in excellent final purities of > 98%.

The following classic comparative in vivo evaluation of the four [^18^F, ^nat^Ga]rhPSMA-7 isomers resulted in less reproducible data sets, and the differences between the biodistribution of each isomer did not allow to select one compound. In addition, there were certain discrepancies between the in vivo and in vitro results. As an example, [^18^F, ^nat^Ga]rhPSMA-7.1 showed the lowest PSMA affinity and the lowest internalization, while its tumor uptake at 1 h p.i. was almost identical to that of [^18^F, ^nat^Ga]rhPSMA-7.3 and -7.4. On the other hand, the more than twofold lower tumor uptake of [^18^F, ^nat^Ga]rhPSMA-7.2 did not correspond with its high PSMA-affinity and internalization rate.

One potential explanation for these results might be differences in the metabolism in individual mice, which become most apparent in experiments involving small animal cohorts (e.g., n = 3 for [^18^F, ^nat^Ga]rhPSMA-7). Furthermore, in vivo degradation of [^18^F, ^nat^Ga]rhPSMA-7 isomers, especially of *L*-Dap-comprising compounds (7.2 and 7.4), by peptidases must be considered [28, 29].

In order to overcome these limitations, we established a radio-HPLC-based analysis to intraindividually determine the differential uptake and excretion pattern of the four isomers with the aim to precisely assess their different in vivo behavior. This method allows to minimize methodological errors, to discover potential metabolites and to reduce the number of experimental animals.

Interestingly, no hydrophilic or lipophilic radiolabeled fragments were detected by radio-HPLC analyses of urine and extracted samples from blood, liver, kidneys and tumor. Due to fewer carboxylic acids and high lipophilicity of the SiFA-moiety (log P = 3.6 [30, 31]), all radioactive species formed by metabolic cleavage should exhibit increased lipophilicity (Additional file 1: Fig. 10). In this context one could argue that potential lipophilic metabolites might not be quantitatively extracted from samples and thus remain unaccounted for. However, such lipophilic species formed in vivo would either bind to plasma proteins and/or show hepatobiliary excretion, resulting in noticeable accumulation in the liver and gastrointestinal system [32, 33]. Due to the high extraction efficiencies in blood (90 ± 4%) and liver (86 ± 2%), such speculations seem unsubstantiated. Moreover, no elevated uptake in these organs was observed in biodistribution studies, indicating high metabolic stability of all isomers. Similarly, defluorination by hydrolysis of the [^18^F]SiFA moiety would have resulted in elevated activity accumulation in bone [34]; again not detected in either biodistribution studies in mice, or in clinical PET scans using [^18^F, ^nat^Ga]rhPSMA-7 [18].

Based on the analysis of the differential uptake and excretion pattern, we favored the *D*-Dap isomers [^18^F, ^nat^Ga]rhPSMA-7.1 and -7.3. These isomers showed higher tumor uptake than *L*-Dap isomers [^18^F, ^nat^Ga]rhPSMA-7.2 and -7.4, which showed lowest accumulation in all analyzed tissues and blood.

Overall, out of the more favorable *D*-Dap-isomers, [^18^F, ^nat^Ga]rhPSMA-7.3 seems to be the isomer of choice, as it shows lower enrichment in blood, liver and kidney, yet still displaying high tumor uptake. In addition, this isomer represented almost ca. 40% of the former diastereomeric mixture [^18^F, ^nat^Ga]rhPSMA-7. Thus, significant problems, such as metabolism, unfavorable organ distribution or tumor uptake in men would have been already detected during the clinical PET investigations with diastereomeric mixture [^18^F, ^nat^Ga]rhPSMA-7.

The intraindividual co-assessment of tracers or isomers, when experimentally feasible, seems to be the evaluation method of choice, as that method takes into account all known or unknown parameters that finally influence the uptake and excretion of a tracer in vivo.

## Conclusion

The analysis of the differential uptake and excretion pattern might be a valuable tool for the preclinical assessment of radiopharmaceuticals. Its potential has been proven in this selection process of [^18^F, ^nat^Ga]rhPSMA-7 isomers where it helped to identify [^18^F, ^nat^Ga]rhPSMA-7.3 as the compound of choice for further clinical development .

## Supplementary information

**Additional file 1**. Supplemental experimental data.

## Data Availability

The datasets used and analyzed during the current study are available from the corresponding author on reasonable request.

## Abbreviations

2-PMPA: 2-(Phosphonomethyl)-pentandioic acid
A_M_: Molar activity
Dap: Diaminopropionic acid
Dde: *N*-1-(4,4-Dimethyl-2,6-dioxocyclohex-1-ylidene)-3-ethyl
DIPEA: Diisopropylethylamine
DOTA-GA: 2-(4,7,10-Tris(carboxymethyl)-1,4,7,10-tetraazacyclododecan-1-yl)pentanedioic acid
Fmoc: Fluorenylmethoxycarbonyl
HATU: (*N*-[(7-Aza-1H-benzotriazol-1-yl)(dimethylamino)-methylene]-*N*-methylmethanaminium-hexafluorophosphate-*N*-oxide
HOBt: 1-Hydroxybenzotriazole
HPLC: High-performance liquid chromatography
HSA: Human serum albumin
ID/g: Injected dose per gram
p.i.: Post injection
PBS: Phosphate-buffered saline
PET: Positron emission tomography
PSMA: Prostate-specific membrane antigen
rh: Radiohybrid
SiFA: Silicon–fluoride–acceptor
SPE: Solid-phase extraction
SPPS: Solid-phase peptide synthesis
TBTU: *N*-[(*1H*-Benzotriazol-1-yl)(dimethylamino)-methylene]-*N*-methylmethanaminium-tetrafluoroborate-*N*-oxide
TFA: Trifluoroacetic acid
TLC: Thin-layer chromatography
