# Supplementary material for: Preclinical comparison of four [18F, natGa]rhPSMA-7 isomers: influence of the stereoconfiguration on pharmacokinetics
Source: EJNMMI Res. 2020 Dec 7;10:149. doi: 10.1186/s13550-020-00740-z (PMC7721954; doi:10.1186/s13550-020-00740-z)
Supplement: Supplementary file 1 — Additional file 1. Supplemental experimental data. [file 13550_2020_740_MOESM1_ESM.docx]

**Preclinical Comparison of four [^18^F, ^nat^Ga]rhPSMA-7 Isomers: Influence of the Stereoconfiguration on Pharmacokinetics**

**– Supporting Information –**

Alexander Wurzer, Mara Parzinger, Matthias Konrad, Roswitha Beck, Thomas Günther, Veronika Felber, Stefanie Färber, Daniel Di Carlo and Hans-Jürgen Wester

Chair of Pharmaceutical Radiochemistry, Technical University of Munich, Garching, Germany

**Corresponding author:**

Alexander Wurzer, PhD

Technical University of Munich
Chair of Pharmaceutical Radiochemistry
Walther-Meißner-Str. 3

85748 Garching

GERMANY

Phone: +49.89.289.12203

Fax: +49.89.289.12204

Email: Alexander.Wurzer@tum.de

# 1. Synthesis of uncomplexed [^19^F]rhPSMA ligands

***[^19^F]rhPSMA-7 (D/L-Dap – R/S-DOTA-GA)***

**Supplemental Figure 1.** Structural formula of the uncomplexed diastereomeric mixture of [^19^F]rhPSMA-7.

Diastereomeric mixture of [^19^F]rhPSMA-7 was prepared as described previously (1). HPLC (10 to 70% B in 15 min): t_R_ = 10.4 min, K’ = 3.73. Calculated monoisotopic mass (C_63_H_99_FN_12_O_25_Si): 1470.7; found: m/z = 1471.8 [M+H]^+^, 736.7 [M+2H]^2+^.

***[^19^F]rhPSMA-7.1 (D-Dap – R-DOTA-GA)***

**Supplemental Figure 2.** Structural formula of uncomplexed [^19^F]rhPSMA-7.1.

The first synthetic steps for preparation of the four different stereoisomers are identical and carried out together, applying the identical synthetic steps as described for [^19^F]rhPSMA-7 (1):

Briefly, resin-bound Fmoc-*D*-Orn(Dde)-OH was Fmoc-deprotected and (*t*BuO)EuE(O*t*Bu)_2_ (2.0 eq.) was conjugated with HOBt (2.0 eq.), TBTU (2.0 eq.) and DIPEA (6.0 eq.) in DMF for 4.5 h. After cleavage of the Dde-group with a mixture of hydrazine in DMF, a solution of succinic anhydride (7.0 eq.) and DIPEA (7.0 eq.) in DMF was added and left to react for 2.5 h. Subsequently, the conjugated succinic acid was pre-activated, by adding a mixture of HOBt (2.0 eq.), TBTU (2.0 eq.) and DIPEA (6.0 eq.) in DMF. After 20 min, Fmoc-*D*-Lys(O*t*Bu)∙HCl (2.0 eq.) dissolved in DMF was added and left to react for 2.5 h. Subsequent cleavage of the Fmoc-group was performed, by adding a mixture of piperidine in DMF. Fmoc-*D*-Dap(Dde)-OH (2.0 eq.) was pre-activated in a mixture of HOBt (2.0 eq.), TBTU (2.0 eq.) and 2,4,6-trimethylpyridine (6.7 eq.) in DMF and added to the resin-bound peptide for 2.5 h. Following orthogonal Dde-deprotection was carried out using imidazole and hydroxylamine hydrochloride dissolved in a mixture of NMP and DMF for 3 h. SiFA-BA (1.5 eq.) was reacted with the free amine of the side chain with HOBt (1.5 eq.), TBTU (1.5 eq.) and DIPEA (4.5 eq.), as activation reagents in DMF for 2 h. After Fmoc-deprotection with piperidine, (*R*)-DOTA-GA(*t*Bu)_4_ (2.0 eq.) was conjugated with HOBt (2.0 eq.), TBTU (2.0 eq.) and 2,4,6-trimethylpyridine (6.7 eq.) in DMF for 2.5 h. Cleavage from the resin with simultaneous deprotection of acid labile protecting groups was performed in TFA for 6 h. After HPLC-based purification, [^19^F]rhPSMA-7.1 was obtained as a colorless solid (19%). HPLC (10 to 70% B in 15 min): t_R_ = 10.4 min, K’ = 3.73. Calculated monoisotopic mass (C_63_H_99_FN_12_O_25_Si): 1470.7; found: m/z = 1471.9 [M+H]^+^, 736.5 [M+2H]^2+^.

***[^19^F]rhPSMA-7.2 (L-Dap – R-DOTA-GA)***

**Supplemental Figure 3.** Structural formula of uncomplexed [^19^F]rhPSMA-7.2.

[^19^F]rhPSMA-7.2 was synthesized as described for [^19^F]rhPSMA-7.1, by conjugation of Fmoc-L-Dap(Dde)-OH and (R)-DOTA-GA(tBu)_4_. [^19^F]rhPSMA-7.2 was obtained as a colorless solid (25%), after HPLC-based purification. HPLC (10 to 70% B in 15 min): t_R_ = 10.3 min, K’ = 3.68. Calculated monoisotopic mass (C_63_H_99_FN_12_O_25_Si): 1470.7; found: m/z = 1471.7 [M+H]^+^, 736.4 [M+2H]^2+^.

***[^19^F]rhPSMA-7.3 (D-Dap – S-DOTA-GA)***

**Supplemental Figure 4.** Structural formula of uncomplexed [^19^F]rhPSMA-7.3.

[^19^F]rhPSMA-7.3 was synthesized as described for [^19^F]rhPSMA-7.1, by conjugation of Fmoc-*D*-Dap(Dde)-OH and (*S*)-DOTA-GA(*t*Bu)_4_. After HPLC-based purification [^19^F]rhPSMA-7.3 was obtained as a colorless solid (35%). HPLC (10 to 70% B in 15 min): t_R_ = 10.3 min, K’ = 3.68. Calculated monoisotopic mass (C_63_H_99_FN_12_O_25_Si): 1470.7; found: m/z = 1471.8 [M+H]^+^, 736.6 [M+2H]^2+^.

***[^19^F]rhPSMA-7.4 (L-Dap – S-DOTA-GA)***

**Supplemental Figure 5.** Structural formula of uncomplexed [^19^F]rhPSMA-7.4.

[^19^F]rhPSMA-7.4 was synthesized as described for [^19^F]rhPSMA-7.1, by conjugation of Fmoc-*L*-Dap(Dde)-OH and (*S*)-DOTA-GA(*t*Bu)_4_. [^19^F]rhPSMA-7.4 was obtained as a colorless solid (27%), after HPLC-based purification. HPLC (10 to 70% B in 15 min): t_R_ = 10.3 min, K’ = 3.68. Calculated monoisotopic mass (C_63_H_99_FN_12_O_25_Si): 1470.7; found: m/z = 1471.9 [M+H]^+^, 736.6 [M+2H]^2+^.

# 2. Synthesis of non-radioactive Gallium complexes

500 µL of a 2 mM stock solution of the uncomplexed [^19^F]rhPSMA ligands (1.0 eq.) in DMSO was combined with 150 µL of a 20 mM Ga(NO_3_)_3_ solution (3.0 eq.) in water. The reaction mixture was heated for 30 min at 75 °C. The complexed compound was purified by HPLC.

***[^19^F, ^nat^Ga]rhPSMA-7:*** HPLC (10 to 70% B in 15 min): t_R_ = 10.4 min, K’ = 3.73. Calculated monoisotopic mass (C_63_H_96_FGaN_12_O_25_Si): 1536.6; found: m/z = 1539.4 [M+H]^+^, 770.3 [M+2H]^2+^.

***[^19^F, ^nat^Ga]rhPSMA-7.1:*** HPLC (10 to 70% B in 15 min): *t_R_* = 10.5 min, K’ = 3.77. HPLC (25 to 35% B in 40 min): *t_R_* = 31.4 min, K’ = 13.27. Calculated monoisotopic mass (C_63_H_96_FGaN_12_O_25_Si): 1536.6; found: m/z = 1539.4 [M+H]^+^, 770.3 [M+2H]^2+^.

***[^19^F, ^nat^Ga]rhPSMA-7.2:*** HPLC (10 to 70% B in 15 min): t_R_ = 10.4 min, K’ = 3.73. HPLC (25 to 35% B in 40 min): t_R_ = 27.9 min, K’ = 11.68. Calculated monoisotopic mass (C_63_H_96_FGaN_12_O_25_Si): 1536.6; found: m/z = 1539.2 [M+H]^+^, 770.2 [M+2H]^2+^.

***[^19^F, ^nat^Ga]rhPSMA-7.3:*** HPLC (10 to 70% B in 15 min): *t_R_* = 10.4 min, K’ = 3.73. HPLC (25 to 35% B in 40 min): *t_R_* = 28.1 min, K’ = 11.77. Calculated monoisotopic mass (C_63_H_96_FGaN_12_O_25_Si): 1536.6; found: m/z = 1539.0 [M+H]^+^, 770.1 [M+2H]^2+^.

***[^19^F, ^nat^Ga]rhPSMA-7.4:*** HPLC (10 to 70% B in 15 min): *t_R_* = 10.5 min, K’ = 3.77. HPLC (25 to 35% B in 40 min): *t_R_* = 29.1 min, K’ = 12.23. Calculated monoisotopic mass (C_63_H_96_FGaN_12_O_25_Si): 1536.6; found: m/z = 1539.1 [M+H]^+^, 770.2 [M+2H]^2+^.

**3. Automated Synthesis of [^18^F, ^nat^Ga]rhPSMA-7**

The automated production was performed, employing a double-cassette setup (Supplemental Figure 6). In a first step, aqueous [^18^F]fluoride (up to 100 GBq) in ^18^O-enriched target water (2.5 mL) is drawn under vacuum on the QMA cartridge (Sep-Pak Accell Plus QMA Carbonate Plus Light cartridge, 46 mg, 40 μm, Waters), preconditioned with 10 mL water and attached to valve 2. Thereafter the QMA is flushed with air, followed by 10 mL of dry MeCN (valve 1) and air. The 2 mL syringe connected to valve 6 contains a solution of the cryptate complex [K^+^⊂2.2.2]OH^−^ (137.5 μmol Kryptofix 222 and 125 μmol KOH ) in 750 μL of MeCN_dry_ which is used for elution of dried [^18^F]fluoride. For the isotopic exchange reaction, the eluate is directly transferred into the reactor, attached to valve 3, which contains 150 nmol of the dissolved [^19^F, ^nat^Ga]rhPSMA-7 precursor (150 μL, 1 mM in DMSO_dry_) mixed with 30 μmol oxalic acid (30 μL, 1 M in MeCN_dry_). After a reaction time of 5 min at rt, the mixture is diluted with 10 mL phosphate buffered saline. For cartridge-based purification an Oasis HLB Plus Short (225 mg sorbent, 60 μm particle size, Waters) is used (valve 4). After passing the diluted reaction mixture through the HLB cartridge, it is flushed with 10 mL of the PBS buffer and dried with air. The purified tracer is then eluted with 3 mL of a 1:1 mixture (*v*/*v*) of ethanol in water (valve 10), followed by 15 mL of PBS buffer and is sterile filtered into the product vial connected at position 5. The production is completed within 16 minutes.


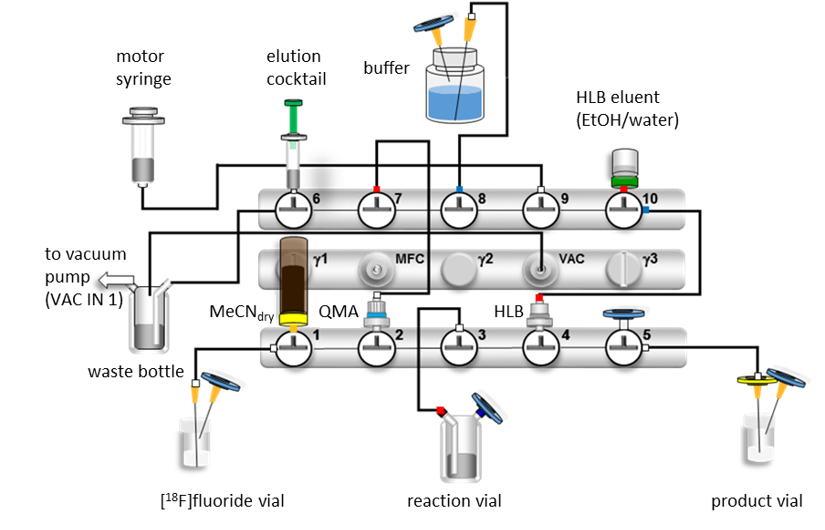


**Supplemental Figure 6.** Set-up of the automatized radiosynthesis of [^18^F, ^nat^Ga]rhPSMA-7.

**4. Differential Uptake and Excretion Pattern**

Processing of the organ samples and body fluids was performed according to the hereafter described procedures. The urine sample was centrifuged for 5 min at 13000*×g* to yield a clear solution and directly subjected to radio-HPLC analysis. Blood was diluted to 1 mL with H_2_O and centrifuged twice at 13000*×*g for 5 min. The supernatant was collected and loaded on a Strata X cartridge (33 μm Polymeric Reversed Phase, 500 mg, Phenomenex, Aschaffenburg, Germany) which was preconditioned with 5 mL MeOH followed by 5 mL H_2_O. After washing with 5 mL of H_2_O, the cartridge was eluted with a 3:2 mixture (*v*/*v*) of MeCN in H_2_O supplemented with 1% TFA. The eluate was diluted with water and analyzed by radio-HPLC. Tumor, kidneys and liver were homogenized using either a Potter-Elvehjem tissue grinder (Kontes Glass, Vineland, United States) or a MM-400 ball mill (Retsch, Haan, Germany):

*Extraction via the Potter-Elvehjem Tissue Grinder.* Tumor and kidneys were separately homogenized in the tissue homogeniser with 1 mL of extraction buffer (850 μL 1 M HEPES pH 7.4, 100 μL 20 mM PMPA and 100 μL 1 M NaCl) for 30 min. The resulting homogenate was collected and centrifuged at 13000*×*g for 5 min. Subsequently, the supernatant was collected, centrifuged again (13000*×*g, 5 min) and loaded on a Strata X cartridge (33 μm Polymeric Reversed Phase 500 mg) preconditioned with 5 mL MeOH, followed by 5 mL H_2_O. After washing with 5 mL H_2_O, the cartridge was eluted with a 3:2 mixture (*v*/*v*) of MeCN in H_2_O supplemented with 1% TFA. For radio-HPLC analysis the eluates were diluted with water.

*Extraction via the MM-400 Ball Mill.* The samples (tumor, kidney, liver) were separately homogenized in a 2 mL tube together with 3 grinding balls (3 mm diameter) and 1 mL of extraction buffer (850 μL 1 M HEPES pH 7.4, 100 μL 20 mM PMPA and 100 μL 1 M NaCl) for 10 min at 30 Hz. The homogenate was centrifuged at 13 000*×*g for 5 min and the supernatant was collected. Subsequently the pellet was suspended in 1 mL of extraction buffer and homogenized again with the ball mill for 10 min at 30 Hz. After centrifugation (13000*×*g, 5 min), both supernatants were combined and loaded on a Strata X cartridge (33 μm Polymeric Reversed Phase 500 mg) preconditioned with 5 mL MeOH followed by 5 mL H_2_O. Following washing and elution steps were performed as described above for the procedure using the Potter-Elvehjem Tissue grinder.

# 5. Supplemental Data

**5.1 HPLC analysis of extracted activity from tissue samples and body fluids**

**Deconvolution and Integration of HPLC chromatograms using PeakFit**


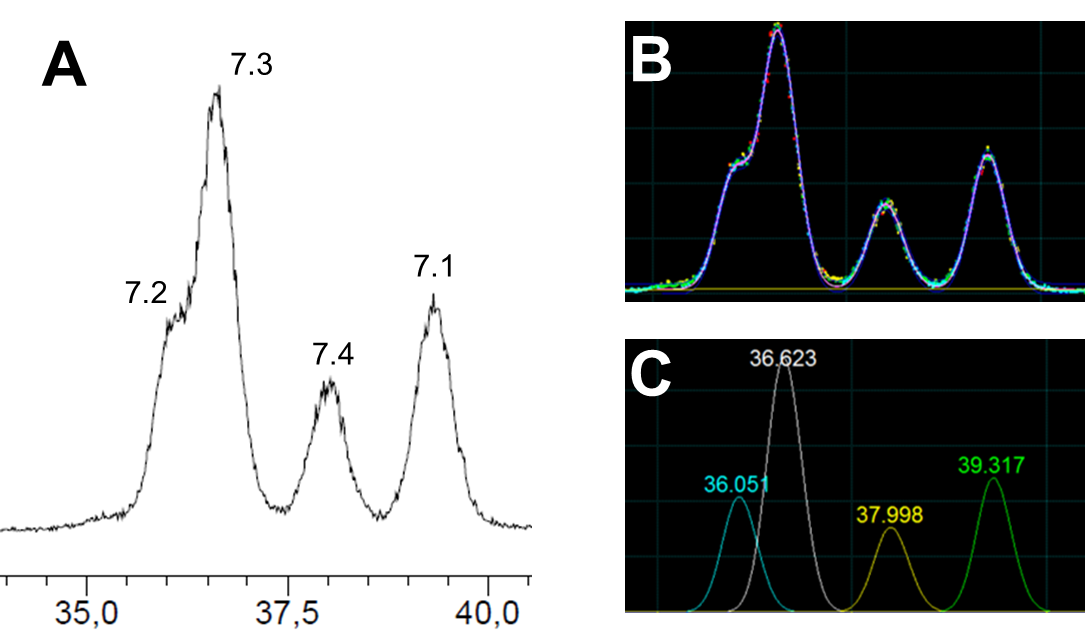


**Supplemental Figure 7. A**) Example of a radio-HPLC analysis of extracted activity from kidneys: extract of the HPLC chromatogram showing [^18^F, ^nat^Ga]rhPSMA-7.1 to -7.4. **B**) Experimental data fitted by the Systat PeakFit Software and **C**) deconvolution into single peaks.

**5.2 Optimization of the HPLC conditions for the quality control of diastereomeric mixture [^19^F, ^nat^Ga]rhPSMA-7**

**
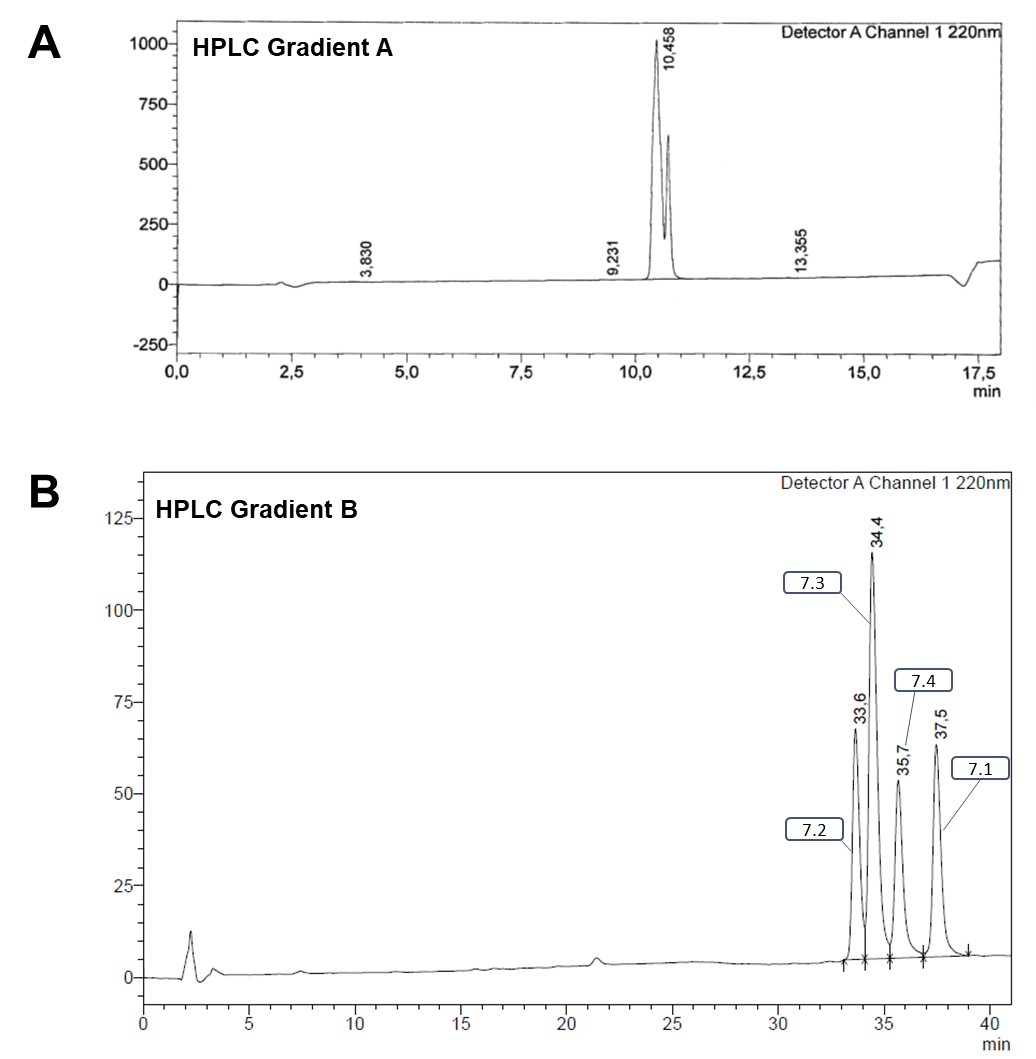
**

**Supplemental Figure 8. A**) Quality control of the diastereomeric mixture [^19^F, ^nat^Ga]rhPSMA-7 using HPLC gradient A (10–70% B 0–15 min, 95% B 15–20 min, 10% B 20–25 min) and **B**) Quality control of the diastereomeric mixture [^19^F, ^nat^Ga]rhPSMA-7 and allocation of isomers [^19^F, ^nat^Ga]rhPSMA-7.1 to -7.4, employing HPLC gradient B (25–35% B 0–40 min, 95% B 40–45 min, 25% B 45–50 min); Solvent A: H_2_O + 0.1% TFA; Solvent B: MeCN + 0.1% TFA, flow: 1 mL/min, column: Nucleosil 100 C18 (125 × 4.6 mm, 5 μm).

**5. 3 In vitro data of PSMA inhibitors**

**Supplemental Table 1.** Binding affinities (IC_50_ in nM, 1 h, 4°C) of [^19^F, ^nat^Ga]rhPSMA-7.1 to -7.4 (n=5-9) and the references diastereomeric mixture [^19^F, ^nat^Ga]rhPSMA-7 (n=3), [^19^F]DCFPyL and [^19^F]PSMA-1007 (n=3); Internalized activity of [^18^F, ^nat^Ga]rhPSMA-7.1 to -7.4 (n=3-6) and the references diastereomeric mixture [^19^F, ^68^Ga]rhPSMA-7 (n=3), [^18^F]DCFPyL and [^18^F]PSMA-1007 (n=3) in LNCaP cells (1 h, 37°C) as a percentage of the reference ligand ([^125^I]I-BA)KuE); Lipophilicity of [^18^F, ^nat^Ga]rhPSMA-7.1 to -7.4 (n=18-23) and the references diastereomeric mixture [^18^F, ^nat^Ga]rhPSMA-7 (n=13), [^18^F]DCFPyL and [^18^F]PSMA-1007 (n=3), expressed as *n*-octanol/PBS (pH 7.4) distribution coefficient (log D); Human serum albumin binding of [^19^F, ^nat^Ga]rhPSMA-7.1 to -7.4 and the references diastereomeric mixture [^19^F, ^nat^Ga]rhPSMA-7, [^19^F]DCFPyL and [^19^F]PSMA-1007, determined on a Chiralpak HSA column. Data for the reference ligands were taken from a previously published studies conducted by our group (1, 2). Values are expressed as mean ± standard deviation.

| **Ligand** | **IC_50_** | **Internalization** | **Lipophilicity** | **HSA-binding** |
| --- | --- | --- | --- | --- |
|  | **[nM]** | **[% of reference]** | **Log D** | **[%]** |
| [^18/19^F, ^nat/68^Ga]rhPSMA-7 | 3.0 ± 0.7 | 126 ± 13 | ­- 3.2 ± 0.1 | ­96 |
| [^18/19^F, ^nat^Ga]rhPSMA-7.1 | 6.9 ± 1.4 | 70 ± 5 | - 3.1 ± 0.3 | ­98 |
| [^18/19^F, ^nat^Ga]rhPSMA-7.2 | 3.7 ± 1.0 | 192 ± 16 | ­- 3.1 ± 0.2 | 98 |
| [^18/19^F, ^nat^Ga]rhPSMA-7.3 | 4.4 ± 1.1 | 161 ± 9 | ­- 3.3 ± 0.2 | 97 |
| [^18/19^F, ^nat^Ga]rhPSMA-7.4 | 3.6 ± 0.7 | 207 ± 4 | ­- 3.3 ± 0.2 | 97 |
| [^18/19^F]DCFPyL | 12.3 ± 1.2 | 118 ± 4 | ­- 3.4 ± 0.03 | 14 |
| [^18/19^F]PSMA-1007 | 4.2 ± 0.5 | 118 ± 5 | ­- 1.6 ± 0.02 | 98 |

**5.4 In vivo data of PSMA inhibitors**

**Supplemental Table 2.** Biodistribution of [^18^F, ^nat^Ga]rhPSMA-7 and the isomers [^18^F, ^nat^Ga]rhPSMA-7.1 to -7.4 at 1 h p.i. in male LNCaP tumor-bearing SCID mice. Data are expressed as a percentage of the injected dose per gram (% ID/g), mean ± standard deviation (n=4 for 7.1, n=5 for 7.2, n=4 for 7.3, n=5 for 7.4 and n=3 for diastereomeric mixture [^18^F, ^nat^Ga]rhPSMA-7).

| **Organ** | **[^18^F, ^nat^Ga] rhPSMA-7** | **[^18^F, ^nat^Ga] rhPSMA-7.1** | **[^18^F, ^nat^Ga] rhPSMA-7.2** | **[^18^F, ^nat^Ga] rhPSMA-7.3** | **[^18^F, ^nat^Ga] rhPSMA-7.4** |
| --- | --- | --- | --- | --- | --- |
| **Blood** | 1.1 ± 0.03 | 0.53 ± 0.13 | 0.56 ± 0.20 | 0.96 ± 0.24 | 1.15 ± 0.30 |
| **Heart** | 0.69 ± 0.07 | 0.53 ± 0.03 | 0.32 ± 0.13 | 0.87 ± 0.17 | 0.71 ± 0.26 |
| **Lung** | 1.4 ± 0.17 | 1.1 ± 0.21 | 0.89 ± 0.38 | 2.2 ± 0.35 | 1.59 ± 0.61 |
| **Liver** | 0.67 ± 0.07 | 0.75 ± 0.62 | 0.35 ± 0.08 | 0.69 ± 0.13 | 0.69 ± 0.20 |
| **Spleen** | 11.1 ± 2.3 | 20.0 ± 4.2 | 10.1 ± 6.3 | 16.6 ± 2.6 | 18.4 ± 9.77 |
| **Pancreas** | 0.60 ± 0.10 | 0.45 ± 0.12 | 0.21 ± 0.08 | 0.63 ± 0.44 | 0.50 ± 0.30 |
| **Stomach** | 0.49 ± 0.07 | 0.28 ± 0.17 | 0.19 ± 0.08 | 0.44 ± 0.23 | 0.25 ± 0.06 |
| **Intestine** | 0.60 ± 0.27 | 0.30 ± 0.16 | 0.18 ± 0.07 | 0.35 ± 0.07 | 0.37 ± 0.09 |
| **Kidneys** | 71.3 ± 13.3 | 220 ± 24.8 | 87.6 ± 28.8 | 292 ± 45.1 | 153 ± 80.3 |
| **Adrenals** | 3.0 ± 0.45 | 2.0 ± 0.25 | 1.3 ± 0.8 | 2.2 ± 0.83 | 3.57 ± 2.38 |
| **Muscle** | 0.36 ± 0.06 | 0.32 ± 0.30 | 0.13 ± 0.07 | 0.33 ± 0.15 | 0.31 ± 0.08 |
| **Bone** | 0.91 ± 0.11 | 0.50 ± 0.31 | 0.31 ± 0.24 | 0.38 ± 0.32 | 0.62 ± 0.30 |
| **Tumor** | 10.4 ± 0.67 | 14.1 ± 4.1 | 6.5 ± 2.3 | 18.3 ± 7.2 | 18.9 ± 3.27 |

**Supplemental Table 3.** Biodistribution of [^18^F, ^nat^Ga]rhPSMA-7.1 to -7.4, co-injected with 2-PMPA (8 mg/kg) at 1 h p.i. in male LNCaP tumor-bearing SCID mice. Data are expressed as a percentage of the injected dose per gram (% ID/g), mean ± standard deviation (n=3).

| **Organ** | **[^18^F, ^nat^Ga] rhPSMA-7.1** | **[^18^F, ^nat^Ga] rhPSMA-7.2** | **[^18^F, ^nat^Ga] rhPSMA-7.3** | **[^18^F, ^nat^Ga] rhPSMA-7.4** |
| --- | --- | --- | --- | --- |
| **Blood** | 0.86 ± 0.40 | 1.1 ± 0.31 | 0.55 ± 0.14 | 0.82 ± 0.17 |
| **Heart** | 0.37 ± 0.16 | 0.47 ± 0.09 | 0.26 ± 0.04 | 0.37 ± 0.05 |
| **Lung** | 0.85 ± 0.29 | 1.1 ± 0.32 | 0.69 ± 0.10 | 0.74 ± 0.14 |
| **Liver** | 0.43 ± 0.07 | 0.46 ± 0.07 | 0.46 ± 0.14 | 0.48 ± 0.14 |
| **Spleen** | 0.21 ± 0.08 | 0.26 ± 0.07 | 0.35 ± 0.02 | 0.28 ± 0.15 |
| **Pancreas** | 0.16 ± 0.10 | 0.12 ± 0.05 | 0.11 ± 0.02 | 0.18 ± 0.09 |
| **Stomach** | 0.97 ± 0.81 | 0.21 ± 0.06 | 0.76 ± 0.74 | 0.20 ± 0.07 |
| **Intestine** | 0.66 ± 0.32 | 0.33 ± 0.10 | 0.94 ± 0.97 | 0.36 ± 0.08 |
| **Kidneys** | 10.9 ± 2.5 | 10.9 ± 1.0 | 15.5 ± 2.2 | 7.2 ± 2.4 |
| **Adrenals** | 0.003 ± 0.004 | 0.07 ± 0.10 | 0.07 ± 0.09 | 0.03 ± 0.04 |
| **Muscle** | 0.17 ± 0.15 | 0.09 ± 0.03 | 0.09 ± 0.02 | 0.20 ± 0.05 |
| **Bone** | 0.33 ± 0.24 | 0.57 ± 0.39 | 0.34 ± 0.22 | 1.0 ± 0.8 |
| **Tumor** | 0.94 ± 0.22 | 1.0 ± 0.13 | 1.5 ± 0.4 | 0.99 ± 0.19 |

**5.5 Differential Uptake and Excretion Pattern**

**Supplemental Figure 9.** Differential uptake and excretion pattern: relative percentage difference between the abundance of [^18^F, ^nat^Ga]rhPSMA-7.1 to -7.4 in blood, liver, kidney, urine and tumor of male LNCaP tumor-bearing SCID mice (30 min p.i.) compared to the respective abundance found in the quality control of diastereomeric mixture [^18^F, ^nat^Ga]rhPSMA-7. n=4 in four independent experiment; the liver from the first experiment was not analyzed. Values are expressed as mean ± SD.

**
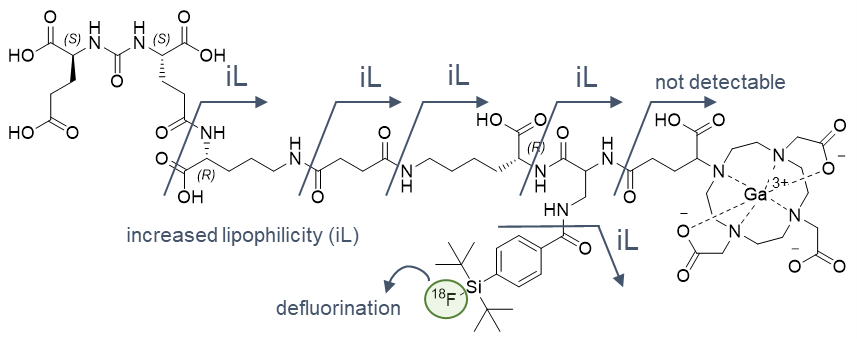
**

**Supplemental Figure 10.** Selection of possibly generated radioactive fragments generated by either metabolic cleavage of amide bonds in [^18^F, ^nat^Ga]rhPSMA-7 or defluorination in vivo. iL: Cleavage forms a species with increased lipophilicity.

# 5. References

1. Wurzer A, Di Carlo D, Schmidt A, Beck R, Eiber M, Schwaiger M, et al. Radiohybrid Ligands: A Novel Tracer Concept Exemplified by (18)F- or (68)Ga-Labeled rhPSMA Inhibitors. Journal of nuclear medicine : official publication, Society of Nuclear Medicine. 2020;61(5):735-42.

2. Robu S, Schmidt A, Eiber M, Schottelius M, Gunther T, Hooshyar Yousefi B, et al. Synthesis and preclinical evaluation of novel (18)F-labeled Glu-urea-Glu-based PSMA inhibitors for prostate cancer imaging: a comparison with (18)F-DCFPyl and (18)F-PSMA-1007. EJNMMI Res. 2018;8(1):30.
